# Recent Trends in Blood Transfusion and Resuscitation

**Published:** 1952-10

**Authors:** Geoffrey H. Tovey

**Affiliations:** Director, South West Regional Blood Transfusion Service; Lecturer in Haematology, University of Bristol


### RECENT TRENDS IN BLOOD TRANSFUSION AND RESUSCITATION

BY

GEOFFREY H. TOVEY, M.D.

*Director, South West Regional Blood Transfusion Service;*
*Lecturer in Haematology, University of Bristol*

The immediate post-war years have witnessed a striking increase in the transfusion of blood and blood products in the hospitals of Great Britain. During 1946, 183,500 pints of blood were issued from the Regional Centres of the National Blood Transfusion Service to hospitals in England and Wales, and in 1951 these issues had risen to 505,000 pints. A similar increase has occurred in the requirements of the three main hospital groups in the Bristol Area, which in 1946 required 4,518 pints, and in 1951, 12,199 pints, an increase of 270 per cent in five years. The greater use of transfusion in medical and surgical practice has been paralleled by advances in the clinical, laboratory and technical aspects of transfusion work, and the more important of these are outlined below.

### CLINICAL ASPECTS

*Acute Haemorrhage and " Wound Shock ".* By means of blood volume studies with the dye T1824, Grant and Reeve (1951) have shown that a diminution in the circulatory blood volume (oligaemia) is the most important factor in the production of wound shock, and that circulatory collapse occurs if a patient's effective blood volume is reduced below 70 per cent of normal. Grant and Reeve conclude that the most valuable signs in determining the degree of reduction in blood volume are the amount of tissue damage, and the systolic blood pressure. They regard the patient's own hand as the unit of measurement, and observed that circulatory blood loss was approximately 10 per cent for each " hand " of tissue damage. Large wounds equivalent to a tissue damage of three to five hands are associated with a 30–50 per cent blood loss, and indicate a transfusion requirement of 3–5 pints in a patient of 10–11 stone. These observations are of particular value as a pointer for transfusion in those exceptional patients who react to haemorrhage with an initial rise in blood pressure, but in the vast majority of patients acute haemorrhage is accompanied by a fall in systolic pressure. Grant and Reeve's findings were that a systolic pressure of less than 100 mm. of mercury indicates a blood volume of less than 70 per cent normal, and they would regard either three " hands" tissue damage or a blood pressure below 100 mm. as indications for immediate transfusion.

Restoration of blood pressure to the patient's normal range must be accomplished without delay if renal tubular damage, cerebral anoxia or " irreversible shock " are to be avoided. In consequence, intra-arterial transfusion is being used for the more rapid correction of oligaemic hypotension. A needle is inserted into the femoral artery, immediately below the inguinal ligament, or if there is severe haemorrhage during an abdominal operation for example, the common iliac artery may be used. Blood is forced rapidly into the arterial tree at a pressure equal to the normal systolic blood pressure. The procedure is likely to be of particular value when haemorrhage is complicated by extreme venous spasm, and some life-saving results have been reported (Bingham 1952). Ischaemic gangrene of the distal portion of the limb used has followed as an occasional complication, but is probably only likely to occur if a cut-down procedure, with cannulation, is employed.

Emphasis is now rightly placed on the importance of adequate pre-operative transfusions in preparing an anaemic patient for a major surgical operation, and there is general agreement that, when correction is practicable, a patient should not undergo operation with the haemoglobin level below 80 per cent of normal (11·8 G. per 100 ml.). Sufficient attention however is not always paid to the fact that anaemia and hypoproteinaemia are accompanied by a compensatory reduction in circulating blood volume (Price 1951), and that pre-operative transfusions should, therefore, be completed well in advance of the day of operation to allow time for circulatory readjustment. For similar reasons, the transfusion of blood at an early stage in the correction of primary or secondary dehydration has proved to be of value, and the haemoglobin level of a patient requiring continuous parenteral fluid therapy should be maintained above 90 per cent (13·3 G. per 100 ml.) by suitable blood transfusion.

Provided constant watch is kept on the patient's blood pressure and there is no engorgement of the neck veins, transfusion cannot be given too rapidly in a case of severe haemorrhage, but over-rapid transfusion is always dangerous in the patient with a severe chronic anaemia. Should the patient's haemoglobin be below 30 per cent (4·4 G. per 100 ml.), the drip rate must not exceed 20 per minute, and 40 drops per minute (one pint in 4 hours) is a safe rate for a transfusion of concentrated red cells to a less severely anaemic patient.

*Exchange or Replacement Transfusion.* Exchange transfusion, replacing pint for pint of the patient's blood with fresh blood, has been given a trial in adults and children with acute leukaemia, following an encouraging preliminary report by Bessis and Bernard (1947). Regrettably, however, such treatment has proved almost entirely disappointing. In some instances there has been a temporary reversion in the blood and marrow picture towards normal following the exchange transfusion. The mechanism underlying this change is worthy of a fuller investigation. Exchange transfusion is also a practical alternative to peritoneal dialysis and the artificial kidney.

The principal application of exchange transfusion however is in the treatment of haemolytic disease of the newborn. A controlled trial, reported by Mollison and Walker (1952), has established that the affected baby treated within 12 hours of birth by an exchange transfusion, to a minimum of 60 ml. per pound body weight, has a significantly greater chance of survival and full recovery than the baby treated by simple transfusion. Of importance also has been the observation during this trial that there was a greater incidence and death rate of kernicterus in babies delivered prematurely than in full-term infants.

*Reactions to Transfusions.* Intense venous spasm may make intravenous transfusion extremely difficult in the " needle-shy " patient or when it complicates severe oligaemia. Repeated intravenous injections of 5–10 ml. of 1 per cent procaine may be followed by sufficient relaxation to enable transfusion to proceed.

Ferris and colleagues (1952) claim that pyrogenic as well as allergic reactions during transfusion can be practically abolished by the routine addition of an anti-histamine (tripelennamine) to each bottle of blood immediately prior to transfusion. The incidence of grade-three pyrogenic reactions following transfusions in Bristol hospitals is as low as one in sixty patients transfused, however, and the risk of complications from the anti-histamine drug itself, together with the additional expense, does not seem to justify its routine employment. Nevertheless, there are certain patients receiving repeated transfusions who react periodically with a rigor for no clear reason, and in whom the measure proposed is worthy of trial.

A striking advance of the post-war period has been the markedly improved outlook for the patient suffering an incompatible transfusion. As a result of the work of Muirhead and his colleagues (1948), Borst (1948), and Bull and others (1949), a patient's chance of recovery following this accident is now at least 90 per cent, whereas five years ago it was variously estimated at between 25 and 40 per cent (Keynes 1947). It is now recognized that (*a*) the extent of the renal tubular damage following an incompatible transfusion can be minimized by the immediate elimination of any concomitant hypotension by the further transfusion of compatible blood or plasma, since sustained hypotension is a well-established cause of acute tubular necrosis, and (*b*) during the phase of renal failure there is a marked, and temporarily irreversible, impairment of excretion of salts and water as well as end-products of metabolism. Treatment during the phase of oliguria or anuria should be based therefore on limiting the patient's water and salt intake to that actually lost by extra-renal plus renal routes, and preventing tissue breakdown by providing an adequate supply of calories in the form of carbohydrates and fats only, thus minimizing the accumulation of urea, potassium, and other toxic products of protein metabolism. Attempts at " flushing through " the patient's kidneys are absolutely contra-indicated as they result in water accumulation and further tubular damage. Alkalis, or saline diuretics, are to be avoided as they lead to sodium retention. Recovery of renal function is accelerated by maintaining the patient's haemoglobin above 70 per cent (10·4 G. per 100 ml.), and penicillin or streptomycin should be given to prevent infection. Detailed accounts of the management of these cases are given by Bull *et al.*, (1949).

Plasma supplied to hospitals in Great Britain during the past four to five years has been prepared from small pools of ten donors only. This has resulted in a reduction in haematogenous hepatitis following plasma transfusion to 1–2 per cent (Lehane *et al.* 1949), whereas the wartime incidence was 10–12 per cent. Experimental evidence has suggested that the causative virus of hepatitis may be destroyed by treating each batch of plasma during preparation with a carefully controlled dose of ultra-violet light, and a clinical trial of U.V.L.-treated plasma is presently being conducted in the Cardiff, Manchester and Liverpool areas.

*Plasma Substitutes.* Each 400 ml. bottle of plasma represents the equivalent of the total contribution of two blood donors, and absolute sterility must be maintained during its drying process, which must be carried out *in vacuo* at a temperature of minus 40°C. Plasma is necessarily therefore an expensive biological material, and is likely always to be available in limited quantity only. Because of this and the risk of subsequent hepatitis in the recipient, there is a continuous search for a satisfactory plasma substitute. Two promising substitutes, dextran and polyvinyl-pyrollidone (PVP) are now being prepared in this country.

Dextran (6 per cent solution in 0·9 per cent sodium chloride) is a polysaccharide synthesised from sucrose by the bacterial activity of *Leuconostoc mesenteroides.* By partial hydrolysis its molecular size can be adjusted to approximate with that of plasma albumin, and consequently the osmotic effects of a dextran transfusion are similar to those following one of plasma. A report on 2,436 pints of dextran transfused to 1,647 patients (Maycock 1952) has shown that the clinical response to dextran transfusion in cases of haemorrhage and burns shock has been very satisfactory. One per cent of patients showed febrile reactions, but these were mostly of mild degree, and nine patients (0·55 per cent) exhibited a mild generalized urticaria. More than 400 pints of dextran have been given in the Bristol hospitals during the past eighteen months, and no serious reactions following their transfusion have to date been reported. Infusion of a large amount of dextran causes a pronounced, and probably undesirable, dilution of plasma-protein as well as dilution of red cells, and, although much of the dextran is eventually excreted in the urine and faeces, it is not yet certain that there is no storage in the tissues. For these reasons it would seem advisable to limit the amount of dextran transfused to two or three pints in patients with haemorrhage and to five or six bottles (correspondingly less in children) in patients with burns, blood or plasma being used to complete the transfusion.

P.V.P. is a synthetic plastic polymer, and a suitable solution giving a colloidal osmotic pressure approximating to that of whole blood is available under the name " Plasmosan ". It does not appear to have been transfused as extensively as dextran, as yet, but preliminary reports (Thrower & Campbell 1951, Arden *et al* 1951) suggest it to be a satisfactory fluid for restoration of blood volume, and no toxic effects have been reported.

### LABORATORY AND TECHNICAL DEVELOPMENTS

*Blood Group Systems.* The therapeutic use of human blood on a scale undreamed of before the war has revealed the multiplicity and complexity of the antigenic components of red blood corpuscles. Nine distinct and genetically unconnected-blood group systems are now recognized, ABO, MNS, P, Rh, Kell, Lutheran, Lewis, Duffy and Kidd. Fortunately, with rare exceptions, only the ABO and the D antigen of the Rh system are of importance to the clinician. The exceptions are virtually limited to those recipients of transfusion who have reacted adversely to a previous blood transfusion and to women who have given birth to children affected with haemolytic disease of the newborn. In such instances the patient may, for example, be " Kell-negative " and have formed immune antibodies type " anti-Kell " as the result of the transfusions or pregnancies. The simpler methods of selecting and matching blood for transfusion will not suffice in the detection of antibodies of this type, and the help of an experienced blood-group reference laboratory needs to be sought if haemolytic reactions to transfusion are to be avoided. Assistance with such difficult cases and the routine serological investigation of expectant mothers have now become two of the important functions of Regional Transfusion Centres. A carefully taken history of previous blood transfusions and of the outcome of any pregnancies is thus an essential accompaniment of a sample of blood for direct matching or ante-natal Rh testing, so that the pathologist may decide whether the test he can apply locally will provide adequate safeguard for the patient or whether a more detailed investigation by a blood-group reference centre is necessary.

A further application of the blood group systems is in cases of disputed paternity. At present the necessary test sera are too scarce to permit the routine employment of all nine sytems, but it has been calculated that using ABO, MNS, Rh, Lutheran, Kell, Duffy and secretion tests, an expert laboratory could exclude about 62 per cent of wrongfully accused persons, whereas by ABO and MN testing alone the chance is only 34 per cent.

*Blood Storage.* Using the present acid-citrate-dextrose anticoagulant and preservative solution, whole blood stored at a constant temperature of between 2° and 6° C. is suitable for transfusion for 21–28 days. The discovery that glycerol largely prevents the death or disruption of living cells when stored at temperatures well below freezing point has opened the way to the prolonged preservation of cells at temperatures where they are in a state of " metabolic arrest ". Experiment has shown that a blood-glycerol mixture can be stored at minus 79° C. for at least nine months, and that if the thawed mixture is freed of glycerol by dialysis about 70 per cent of the red cells are recovered in normal condition (Sloviter 1952). It has yet to be established whether such recovered red cells will survive satisfactorily in human subjects after transfusion, but progress in this field has been sufficiently encouraging to suggest that the prolonged storage of human red cells suitable for subsequent transfusion may in time be practicable.

### REFERENCES

Arden, G. P., Mandow, G. A. and Stoneham, F. J. R. (1951), *Lancet*, **1**, 1099.

Bessis, M., and Bernard, J. (1947) *Bull. Acad. Med.*, Paris, **131**, 615.

Bingham, D. L. C. (1952) *Lancet*, **2**, 157.

Borst, J. G. G. (1948), *Lancet*, **1**, 824.

Bull, G. M., Joekes, A. M., and Lowe, K. G. (1949) *Lancet* **2**, 229.

Ferris, H. E., Alpert, S., and Coakley, C. S. (1952), *Amer. Practit.* **3**, 177.

Grant, R. T., and Reeve, E. B. (1951) Medical Research Council Special Report Series, No. 277, H.M.S.O., London.

Keynes, G. (1949) *Blood Transfusion*, Bristol: J. Wright & Sons Ltd.

Lehane, D., Kwantes, C. M. S., Upward, M. G., and Thomson, D. R. (1949) *Brit. Med. J.* **2**, 572.

Maycock, W. d'A. (1952) *Lancet*, **1**, 1081.

Mollison, P. L., and Walker, W. (1952) *Lancet*, **1**, 429.

Muirhead, E. E., Haley, A. E., Haberman, S., and Hill, J. M. (1948) *Blood*, Special Issue, No. 2, 101.

Sloviter, H. A. (1952) Nature, **169**, 1013.

Thrower, W. R., and Campbell, H. (1951) *Lancet*, **1**, 1096.

